# Structures 4-n-propyl Piperazines as Non-Imidazole Histamine H3 Antagonists

**DOI:** 10.3390/ma14227094

**Published:** 2021-11-22

**Authors:** Andrzej Olczak, Jarosław Sukiennik, Beata Olszewska, Monika Stefaniak, Krzysztof Walczyński, Małgorzata Szczesio

**Affiliations:** 1 Institute of General and Ecological Chemistry, Faculty of Chemistry, Lodz University of Technology, Żeromskiego 116, 90-924 Łódź, Poland; jaroslaw.sukiennik@dokt.p.lodz.pl; 2 Department of Synthesis and Technology of Drugs, Medical University, Muszyńskiego Street 1, 90-145 Łódź, Poland; beata.olszewska@umed.lodz.pl (B.O.); monika.stefaniak@umed.lodz.pl (M.S.); krzysztof.walczynski@umed.lodz.pl (K.W.)

**Keywords:** crystal structure, non-imidazole histamine H3 antagonists

## Abstract

Seven new low-temperature structures of 4-n-propylpiperazine derivatives, potential H3 receptor antagonists, have been determined by X-ray crystallography, with the following symmetry and unit cell parameters: 2-(4-propyl-piperazin-1-yl)oxazolo[4,5-c]pyridine (compound 1), *P*-1, 5.9496 Å, 12.4570 Å, 12.8656 Å, 112.445°, 95.687°, 103.040°; 2-(4-propyl-piperazin-1-yl)thia-zolo[4,5-c]pyridine (compound 2), *I*2/*a,* 22.2087 Å, 7.5519 Å, 19.9225 Å, β = 92.368°; 2-(4-propyl-piperazin-1-yl)oxazolo[5,4-c]pyridine (compound 3), *C*2/*c,* 51.1351 Å, 9.36026 Å, 7.19352 Å, β = 93.882°; 2-(4-propyl-piperazin-1-yl)thiazolo[5,4-c]pyridine (compound 4), *Pbcn*, 19.2189 Å, 20.6172 Å, 7.4439 Å; 2-(4-propylpiperazin-1-yl)[1,3]oxazolo[4,5-b]pyridine, hydrate (structure 5), *Pbca*, 7.4967 Å, 12.2531 Å, 36.9527 Å; 2-(4-propylpiperazin-1-yl)[1,3]oxazolo[4,5-b]pyridine, first polymorph (structure 6), *P*-1, 7.2634 Å, 11.1261 Å, 18.5460 Å, 80.561°, 80.848°, 76.840°; 2-(4-propylpiperazin-1-yl)[1,3]oxazolo[4,5-b]pyridine, second polymorph (structure 7), *P*2_1_, 8.10852 Å, 7.06025 Å, 12.41650 Å, β = 92.2991°. All the compounds crystallized out as hydrobromides. Oxazole structures show a much greater tendency to form twin crystals than thiazole structures. All the investigated structures display N—H···Br hydrogen bonding. (ADME) analysis, including the assessment of absorption, distribution, metabolism, and excretion, determined the physicochemical properties, pharmacokinetics, drug similarity, and bioavailability radar, and confirmed the usefulness of the compounds in question for pharmaceutical utility. This work is a continuation of the research searching for a new lead of non-imidazole histamine H3 receptor antagonists.

## 1. Introduction

The histamine H3 receptor has been the subject of much recent interest, due to its central role in regulating neurotransmitter levels. This G-protein-coupled receptor acts as presynaptic auto- and heteroreceptor, mainly in the central nervous system (CNS) [1], controlling the synthesis and release of histamine, but also modulating several other neurotransmitter systems, e.g., acetylcholine [2,3], dopamine [4], noradrenalin [5], and serotonin [6]. A variety of potential therapeutic applications for H3 receptor antagonists/inverse agonists have been proposed as potential drugs for the treatment of several CNS disorders, such as attention-deficit hyperactivity disorder (ADHD) [7], Alzheimer’s disease [8], and schizophrenia [9]. The physiological and pathophysiological implications of histamine H3 receptors increase the need for potent and selective ligands as pharmacological tools and for potential drug development. The first generation of H3 antagonists was characterized by the presence of an imidazole ring as in histamine, many of which have found utility as pharmacological tools [10]. In contrast to the early work in the field, most chemical series of current interest appear to be non-imidazole compounds because of the major disadvantages of the 4-substituted imidazole moiety, including poor brain penetration and issues related to hepatic cytochrome P450 enzyme inhibition, such as drug–drug interactions, liver toxicity, and inhibition of adrenal synthesis [11].

**Figure 1:**
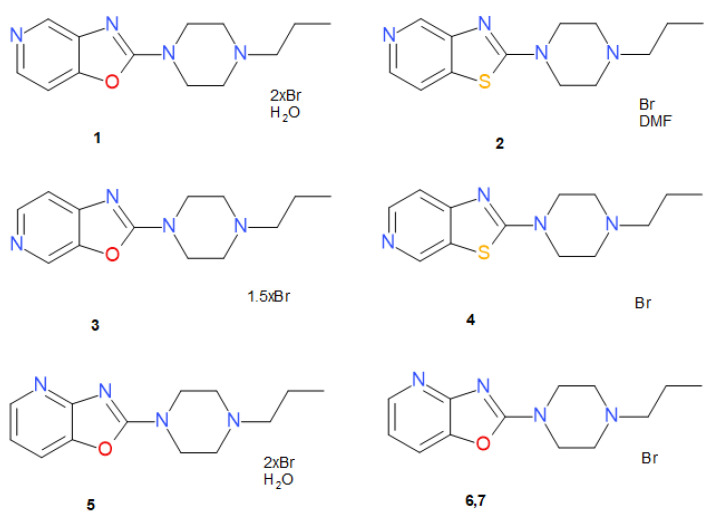
Structural formulas of studied compounds.

Some years ago, we reported the synthesis and pharmacological characterization of the series of 2-(4-propylpiperazin-1-yl)thiazolopyridines and their analogue 2-(4-propylpiperazin-1-yl)-oxazolopyridines in vitro. Thiazoles displayed a higher activity than their oxazole analogs [12]. The most active compounds of both series are presented in Figure 1.

The rational design of new highly active compounds that selectively bind to specific receptors requires both knowledge of the active site structure of the receptor and the ligand itself. The compound represented by structures 5, 6, and 7, having no significant activity, was added for comparative purposes.

In the present study, we report the crystallographic investigation of the following seven new structures **1**–**7**: (2-(4-propylpiperazin-1-yl)oxazolo[4,5-c]pyridine—compound **1**; 2-(4-propylpiperazin-1-yl)thiazolo[4,5-c]pyridine—compound **2**; 2-(4-propylpiperazin-1-yl)oxazolo[5,4-c]pyridine—compound **3**; 2-(4-propylpiperazin-1-yl)thiazolo[5,4-c]pyridine—compound **4**; 2-(4-propylpiperazin-1-yl)[1,3]oxazolo[4,5-b]pyridine crystallized as two polymorphs (**6**, **7**) and a hydrate (**5**) (Figure 1). The obtained data can be used for molecular modeling of these ligands, e.g., in calculations of docking to the receptor, provided the receptor structure is known, or in 3D QSAR or pharmacophore design, when more structures of this type are identified.

## 2. Materials and Methods

The synthesis of all compounds was described by Walczyński [12]. Single crystals of compounds 1, 2, 3, 4, 5, 6 and 7, suitable for X-ray diffraction, were obtained from methanol–water–DMF (1:1:1 *v*/*v*) solutions by slow evaporation of the solvents at room temperature. The crystal measurements were performed on a XtaLAB Synergy diffractometer, Dualflex, Pilatus (Bioz Stars, Los Altos, CA, USA) 300 K [13]. All diffraction experiments were carried out with CuKα radiation. Diffraction data were processed with CrysAlis PRO (Rigaku Oxford Diffraction. CrysAlis PRO; Rigaku Oxford Diffraction Ltd: Yarnton, Oxfordshire, England, 2020) [14]. Crystal structure solution and refinement were carried out with SHELX [15,16]. All H atoms (except majority of those engaged in hydrogen bonds) were geometrically optimized and allowed as riding atoms, with C—H = 0.95 Å for aromatic CH groups, 0.97 Å for secondary CH_2_ groups and 0.96 Å for methyl groups, and N—H = 0.86 Å, with U_iso_(H) = 1.2 U_eq_(C, N). In all studied structures, the methyl H atoms were refined with U_iso_(H) = 1.5 U_eq_(C).

Quantum calculations were performed with GAMESS-US quantum computing package [17] using DFT/B3LYP [18,19,20,21] functional with the base functions 6-311 G(d,p) to optimize the geometry of the studied compounds, taking into account the solvent effect of water using polarizable continuum model. The MOLDEN package [22] was used for the preparation of input files and for visualization purposes. MULTIWFN was used for calculation of CHELPG charges [23].

Additionally, ADME analysis was performed to test the use of the studied compounds as drugs. ADME analysis was performed using SwissADME service (Swiss Institute of Bioinformatics 2021) [24,25,26] and ProTOX II service to predict toxicities of tested compounds [27].

## 3. Results and Discussion

### 3.1. Crystallography

The crystal data, data collection, and structure refinement details are summarized in Table 1 (full details are deposited in supplementary materials).

Except structure **7**, all the oxazole structures revealed a propensity for twinning. For structure **1**, two components of the crystal were rotated approximately 2°, and were difficult to separate, hence the data were processed as a single crystal, giving rise to high mosaicity. For structure **3**, many components were identified in all the studied crystals. Finally, a crystal with four components was chosen for the diffraction experiment. The contributions of the components to the total intensity were the following: 0.51, 0.23, 0.02, and 0.24. Moreover, in this structure, very high disorder for Br anions, and probably DMF and water molecules, was observed, which could not be resolved, and, consequently, the SQUEEZE procedure [28] has been applied. For structure **5,** four components were also identified, with the following contributions to the total intensity: 0.37, 0.09, 0.25, and 0.29. For structure 6, only two components were identified, with contributions of 0.58 and 0.42.

**Figure 2:**
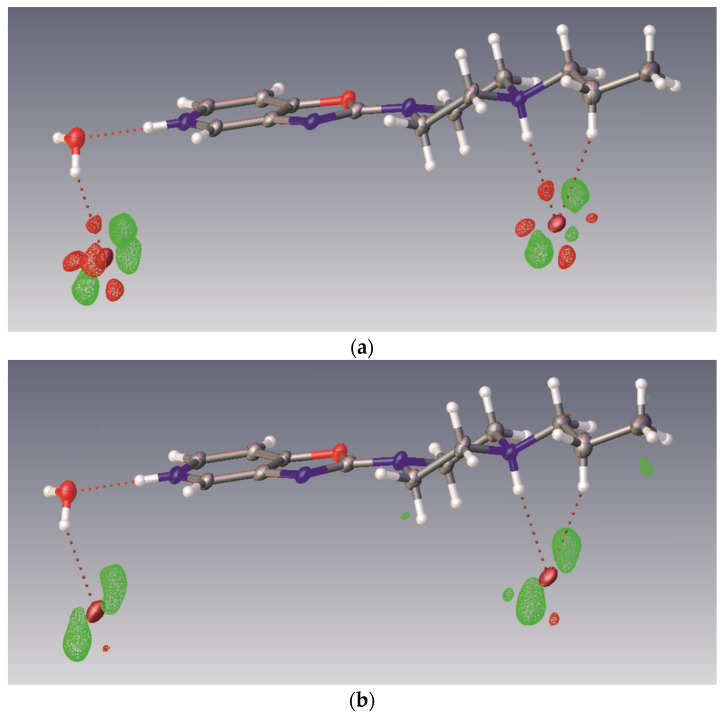
Difference Fourier electron density maps for 1. (**a**) Harmonic approximation to displacement factors for non-hydrogen atoms resulting in Δρ_max_ = 1.6 and Δρ_min_ = 1.0 (eÅ^−3^). (**b**) Anharmonic approximation to the Br ion displacements taking into account 10 components of C_ijk_ and 15 components of D_ijkl_ symmetric tensors resulting in Δρ_max_ = 1.1 and Δρ_min_ = 0.4 (eÅ^−3^). Drawing prepared with Olex2 v.1.3.0 [29].

In all the oxazole structures, high values of residual electron density were located in the difference electron density maps in the vicinity of Br anions (Table 1, Figure 2a). This is probably (at least partly) due to the anharmonicity of the thermal motion of the ions, because taking into account the third and fourth orders of displacement parameters (25 additional parameters) (Olex2 v.1.3.0) [29] leads to a significant reduction in the residual maxima for structure **1** (from 1.6 to 1.1 e/A^3^, and from −1.0 to −0.4 e/A^3^) (Figure 2b), and to a reduction in the R factor from 4.2% to 3.5%, with a still relatively high N_ref_/N_par_ ratio of 14. Similar behavior is observed for, e.g., structure **7,** where the reduction in the residual maxima amounts to 1.4 e/A^3^ (from 2.2 to 0.8 e/A^3^) and the reduction in the R factor from 3.3% to 2.1%, with an N_ref_/N_par_ ratio of 15. On the contrary, for thiazole derivatives (**2**, **4**), such anomalies in the difference Fourier maps were not observed.

**Figure 3:**
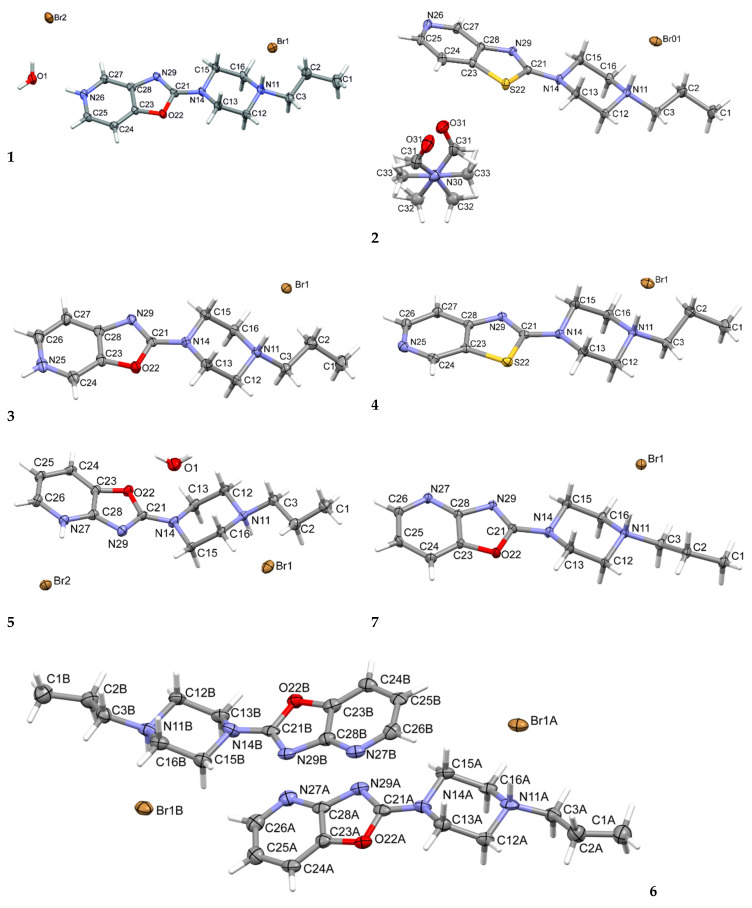
The molecular structures of **1**, **2**, **3**, **4**, **5**, **6** and **7**, showing the atom-labeling schemes. Displacement ellipsoids are drawn at the 50% probability level and H atoms are shown as small spheres of arbitrary radii. Drawing prepared with Mercury software [30].

The molecular structures of the studied compounds in their crystals are shown in Figure 3.

The structures **2**, **4**, **6,** and **7** crystallized out in the monoprotonated form. In all the investigated compounds, the piperazine nitrogen atom N11 is protonated. Compound **2** crystallizes as a solvate, with a disordered DMF molecule built into the structure. In contrast to the **2**, **4**, **6,** and **7** compounds, **1** and **5** crystallized out as a diprotonated hydrate.

**Figure 4:**
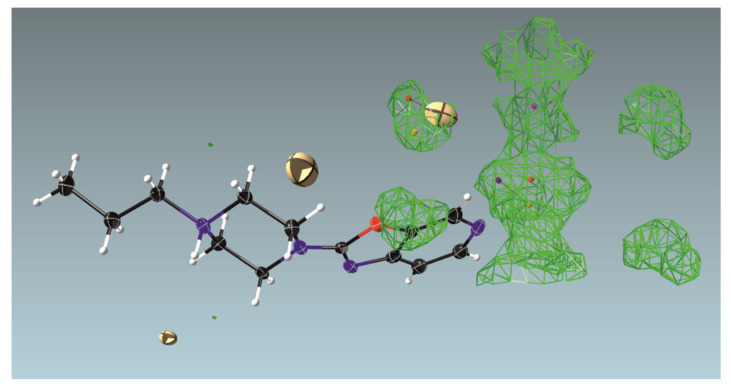
Difference electron density map for 3 showing the area of electron density representing delocalized solvent molecules (probably DMF and water) and probable sites of disordered bromide anions (two big yellow ellipsoids), which were excluded from the refinement and instead SQEEZE procedure was applied. Drawing prepared with ShelXle.

For structure **3,** very high structural disorder is observed. Large maxima in the difference Fourier map with electron densities of ~19.2 and 11.2 e/A^3^ (Figure 4) can be attributed to sites partially occupied by bromide anions, with displacement factors ~3 times higher than that for the Br1 anion. The remaining maxima seem to be uninterpretable.

**Figure 5:**
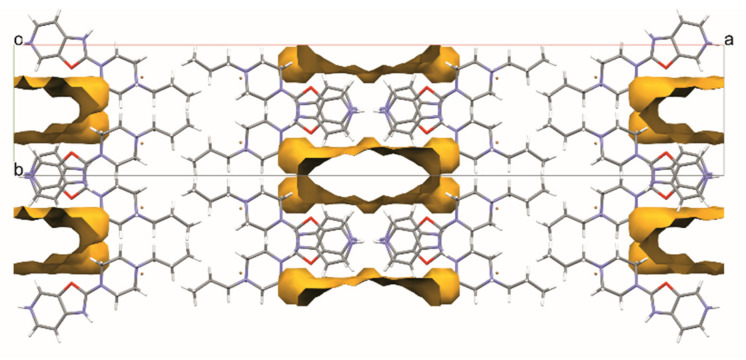
Voids identified by SQUEEZE procedure in structure 3. The voids contain disordered Br anions and possibly DMF and water molecules. The voids form channels across the crystal structure down the *c* axis.

Omitting this whole part of the structure, one can observe large voids forming channels throughout the crystal structure along the [001] direction (Figure 5). Such channel-like structures may lead to high disorder that is difficult to resolve, which is why we decided to use the SQUEEZE procedure in PLATON. The resulting voids constitute approximately 18% of the volume of the unit cell, and there were 149 recovered electrons in a single void. As we can observe, e.g., for structures **1**, **2,** or **5,** both water and DMF molecules can be built into the structure of the studied compounds. Hence, we assume that there are two bromide anions in each channel, one DMF, and four water molecules, which gives 150 electrons per void.

Thus, it seems that this structure contains 1.5 bromide anions per molecule with the protonated piperazine nitrogen atom N11 and the half-protonated nitrogen atom N25, which ensures that charge balance is preserved. Hence, the crystal structure is a 1:1 mixture of mono-cations and di-cations of compound **3**. The disordered Br^−^ anion (1/2 per molecule of **3**) and solvent molecules (0.25 DMF per molecule and 1 H_2_O per molecule) are located in the channel of the blur of electron density (Figure 4).

**Figure 6:**
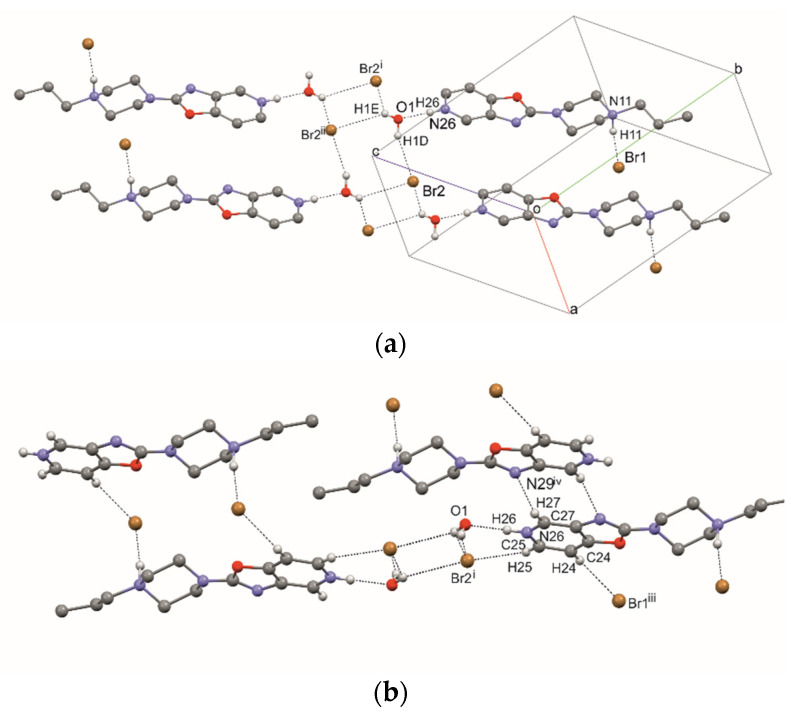
The intermolecular hydrogen bonds in compound **1**. (**a**) Strong hydrogen bonds, (**b**) all hydrogen bonds. [Symmetry code: (i) x−1, y, z; (ii) −x, −y, −z+2; (iii) −x, −y+1, −z+1; (iv) −x+1, −y+1, −z+2.].

**Figure 7:**
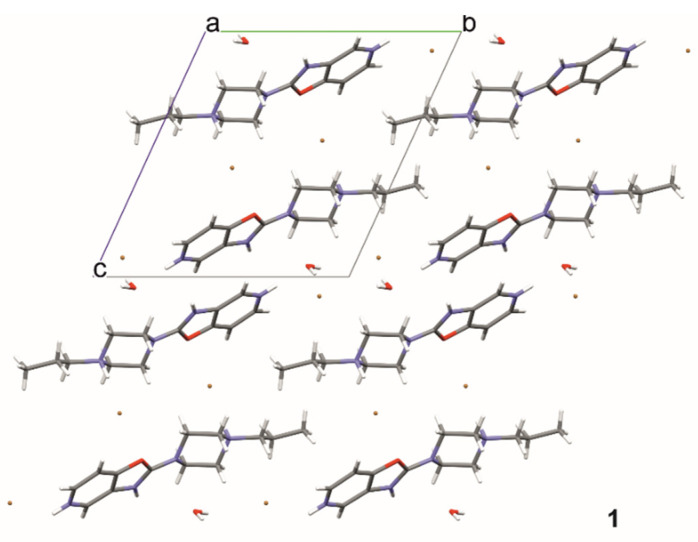
The crystal packing of **1**, viewed along the a-axis, where a, b and c denote unit cell axes.

In structure **1,** ribbons are formed by hydrogen bonds of the studied molecule with water molecules and Br2 (C1,2(4) symbol—according to the graph-set theory of Bernstein [31]—Figure 6, Table 2). These ribbons are joined together by hydrogen bonding, N11—H11 ··· Br1 and C24—H24 ··· Br1 (R2,4(22)), and weaker bonds to C27—H27 ··· N29 (R2,2(8). The stacking interaction (3.241(1) Å) additionally stabilizes the layered packing of the molecules. Water molecules and bromide ions fill the gaps between the molecules of compound **1,** and they line up along the [100] direction (Figure 7).

**Figure 8:**
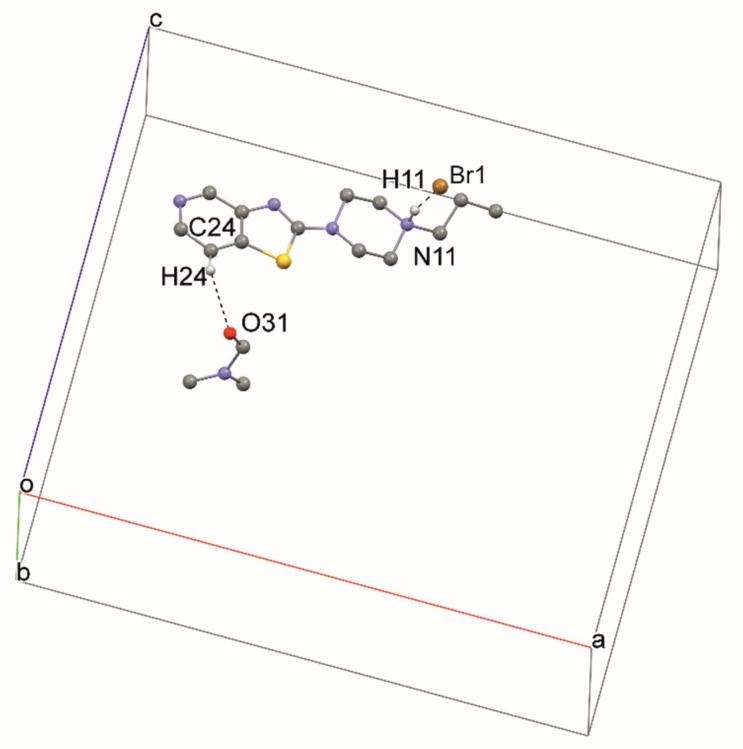
The intermolecular hydrogen bonds in compound **2**.

**Figure 9:**
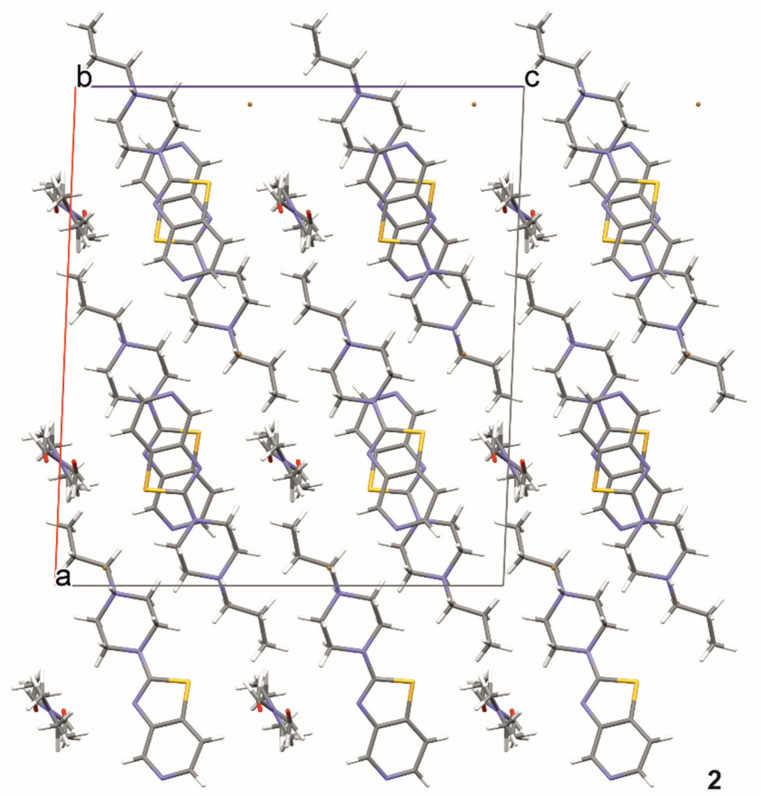
The crystal packing of **2**.

In structure **2**, a strong hydrogen bond, N11—H11···Br1, is formed (Figure 8, Table 3). Additionally, the molecule of compound **2** forms a hydrogen bond, C24—H24···O31, with the DMF molecule. DMF molecules fill the channel formed along the [010] direction (Figure 9).

**Figure 10:**
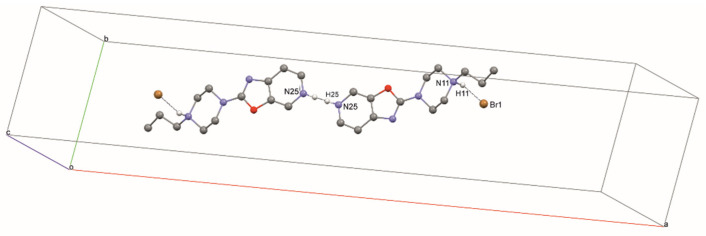
The intermolecular hydrogen bonds in compound **3**.

**Figure 11:**
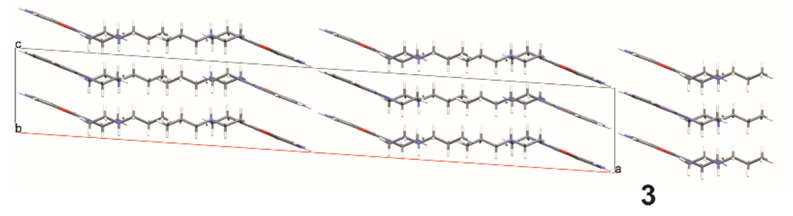
The crystal packing of **3**, viewed along the b-axis.

Additionally, in structure **3**, a typical hydrogen bond, N11—H11···Br1, is present. In addition, there is an interesting hydrogen bond, N25—H25···N25, forming a dimer (Figure 10, Table 4) where the H25 atom site is half occupied. The stacking interaction (3.346 (1) Å) stabilizes the layered packing of the molecules (Figure 11).

**Figure 12:**
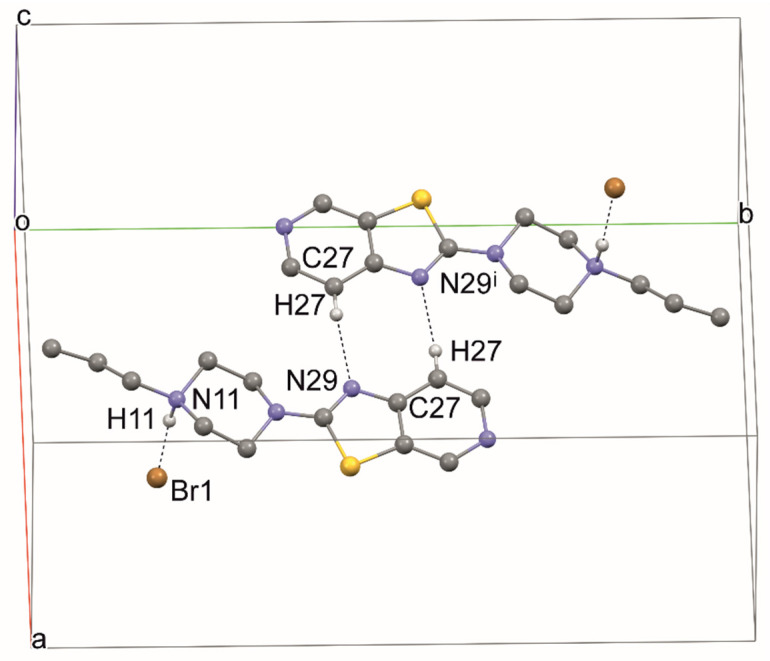
The intermolecular hydrogen bonds in compound **4**.

**Figure 13:**
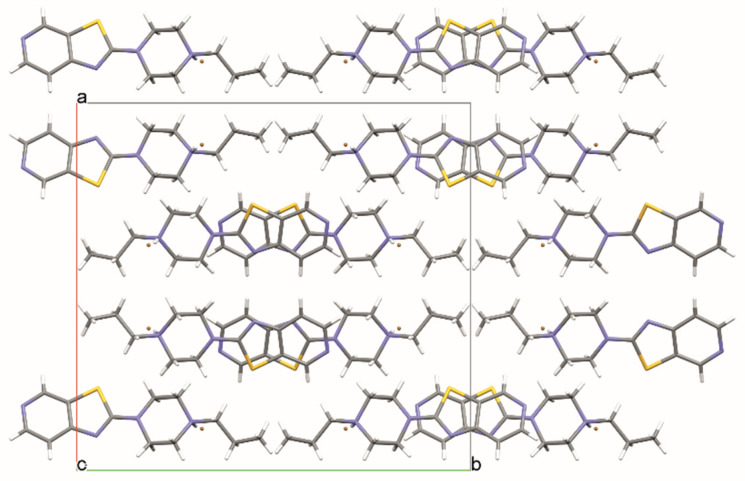
The crystal packing of **4**.

As in all the above structures, the typical hydrogen bond N11—H11 ··· Br1 is also present in structure 4. Moreover, a dimer is formed through C27—H27···N29 hydrogen contacts (forming ring R2,2(8)) (Table 5, Figure 12). The layered system is stabilized by stacking interactions at a distance of 3.452 (1) Å (Figure 13).

**Figure 14:**
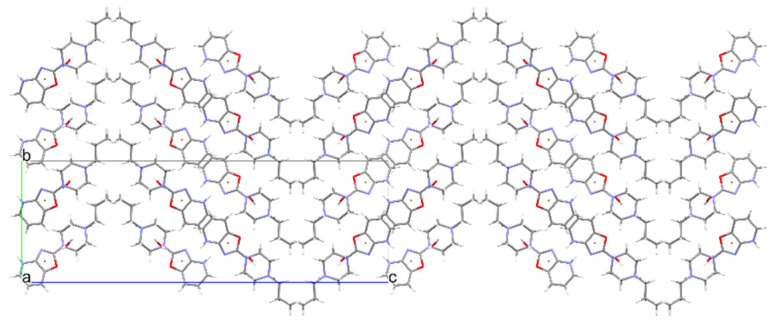
The crystal packing of **5**.

**Figure 15:**
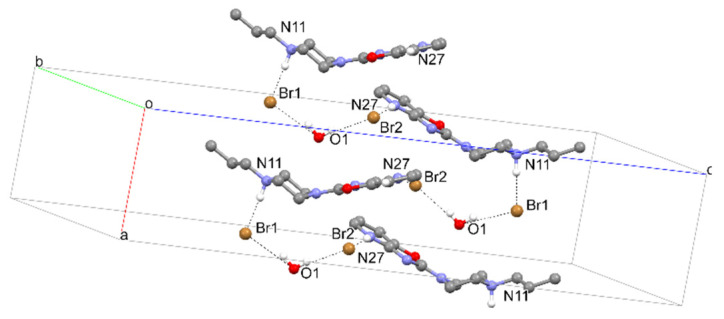
The intermolecular hydrogen bonds in compound **5**.

Structures **5**, **6**, and **7** were obtained from the same sample. Structure **5** is in the form of a dibromohydrate. The packing of this structure differs from the analogous dibromohydrate (compound **1**) (Figure 14). In this case, all the strong hydrogen bonds (Table 6 and Figure 15) form a chain. Additionally, the weak hydrogen bonds (C–H…O, Br, or N type) stabilize the packing of the molecules.

**Figure 16:**
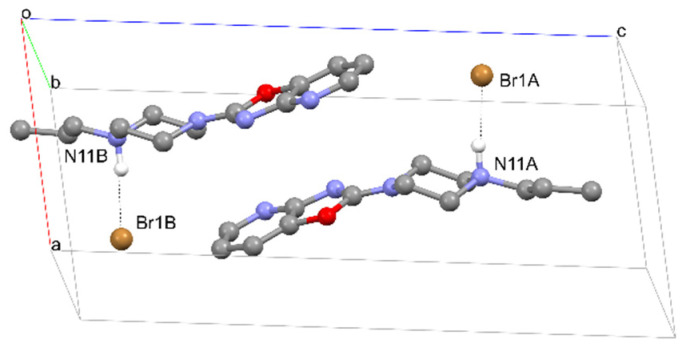
The intermolecular hydrogen bonds in compound **6**.

**Figure 17:**
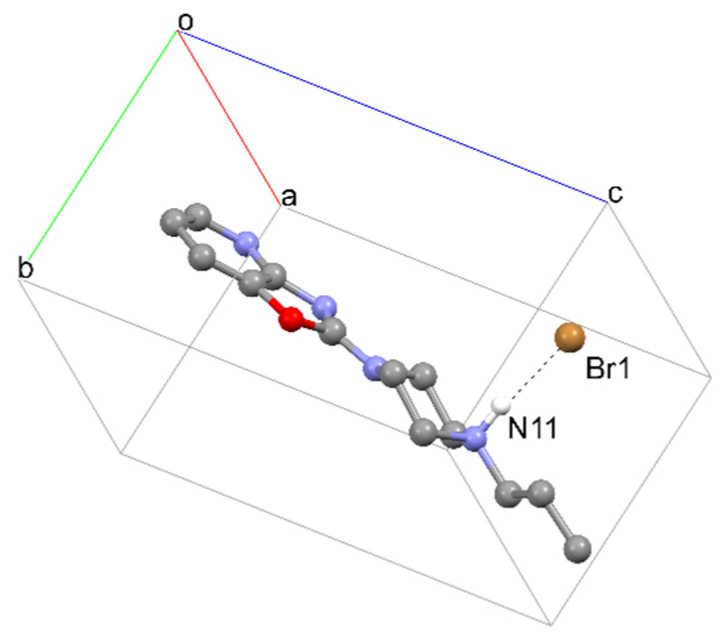
The intermolecular hydrogen bonds in compound **7**.

**Figure 18:**
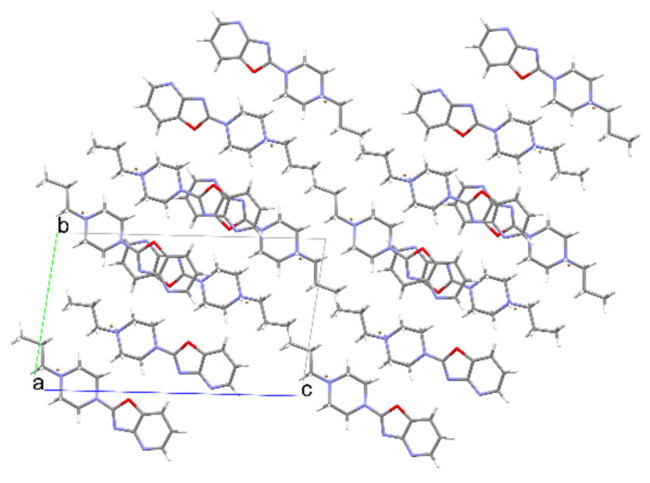
The crystal packing of **6**.

**Figure 19:**
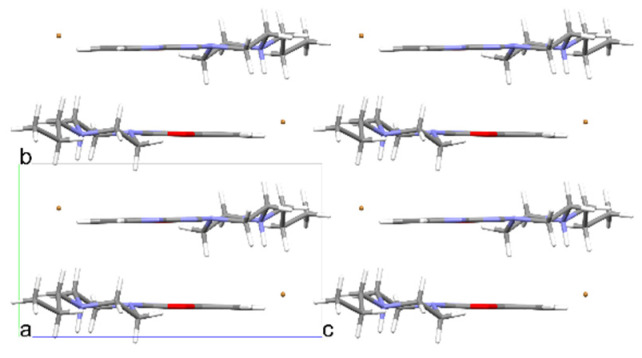
The crystal packing of **7**.

Structures **6** and **7**, on the other hand, are polymorphs of this compound. Both of these structures are in the form of bromide, and have the same strong hydrogen bond between bromine and the piperazine system (Br…H–N) (Figure 16 and Figure 17, and Table 7 and Table 8). Analysis of molecular packing (Figure 18 and Figure 19) reveals different polymorphs. In structure **7**, a layered arrangement of molecules is observed (Figure 19).

**Figure 20:**
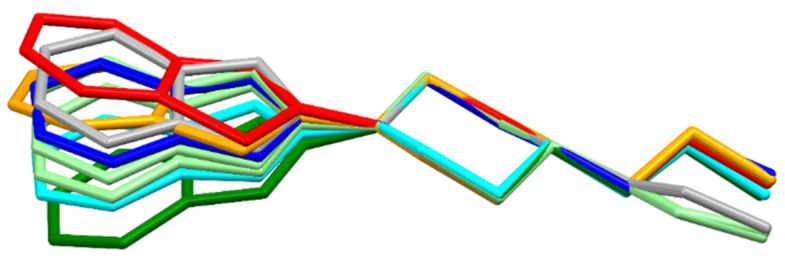
Overlay of molecules for all determined structures; **1**—blue; **2**—red; **3**—cyan; **4**—orange; **5**—green; **6**—light green; **7**—grey.

The greatest variation in the geometry of the studied molecules can be observed on the nitrogen atom N14, the conformation of which determines the direction of the two-ring aromatic system (Figure 20). This conformation can be defined as the angle, let us call it δ, between the N14—C21 bond and the plane formed by the following atoms: C13, N14, and C15. For the most planar case (structure **5**), the angle is less than 1°, and the highest value of this angle, almost 30°, is for structure **2**. The δ angle takes the following values for the individual structures: **1**—23.07°; **2**—29.38°; **3**—10.22°; **4**—20.99°; **5**—0.74°; **6**A—17.34°; **6**B—23.84°; **7**—27.91°.

Another clear conformational difference shown in Figure 20 is the conformation of the aliphatic chain, which can be described by the C2–C3–N11–C16 torsion angle. This angle takes the following values for the individual molecules: **1**—61.7 (0.4); **2**—63.8 (0.2); **3**—62.7 (0.3); **4**—70.3 (0.2); **5**—56.7 (0.8); **6**A—−177.3 (0.7); **6**B—178.1 (0.6); **7**—167.7 (0.3).

**Figure 21:**
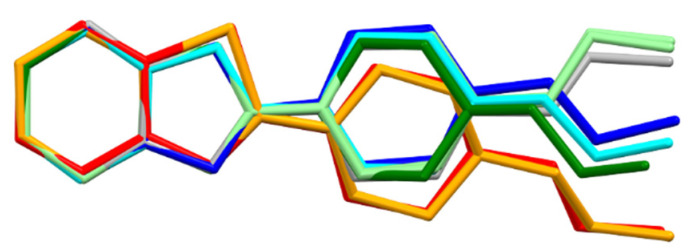
Overlay of molecules for all determined structures; **1** is blue, **2** is red, **3** is cyan, **4** is orange, **5** is green, **6** is light green and **7** is grey.

The structural feature that clearly distinguishes thiazole derivatives from oxazole derivatives is the geometry of the five-membered ring. This geometry, in turn, determines the position of a piperazine ring (Figure 21), which can influence the biological activity of the compounds. It is known that, among the studied structures, the thiazoles are more active than the oxazoles [12].

### 3.2. Density Functional Theory (DFT) Calculations

We were interested in how many conformations of the studied molecules in their crystal environment were different from those optimized in solution, where molecules experience more conformational freedom, to observe how the interactions in the crystal modify these conformations. For calculations, we used the GAMESS-US quantum computing package with the parametrization described in the Materials and Methods section.

It turned out that the optimized conformations were very close to the crystallographic conformations for all the examined structures. Thus, it seems that the degree of freedom associated with the above-mentioned angle δ is very “soft”, and, therefore, its variability among the examined structures is most likely due to the intermolecular interactions in the crystalline state. The other parameters determining the geometry of the optimized molecules are also very similar to those observed in crystals; hence, in the case of the tested compounds, crystallographic studies provide structural information that can be transferred directly to the non-crystalline state.

**Figure 22:**
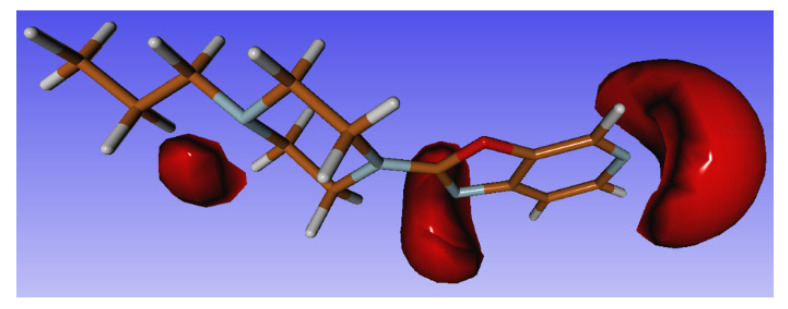
Isosurface of negative (−0.3) electrostatic potential calculated with *MOLDEN* (option: true electrostatic potential) for neutral molecule **3** in optimized geometry.

Using the geometry of molecule **3** as a starting point, we optimized its geometry in an unprotonated form, and calculated the electrostatic potential (ESP) charges with the MULTIWFN [23] package for this unprotonated molecule, to see which of the four nitrogen atoms is most willing to attach protons (Table 9). In Figure 22, a graphical representation of the electrostatic potential is presented.

Unfortunately, these results do not correlate well with the structures determined experimentally, where the first protonation candidate is the N11 atom, followed by the N25 atom. Moreover, protonation of the N29 atom was not observed in any of the experimentally determined structures, which, according to ESP calculations, is best suited for the attachment of a proton.

We also calculated the free energies of the molecules in the mono-protonated state, for all the nitrogen atoms, by the PCM method, with water as a solvent. The results are presented in Table 9. The lowest energy occurs for N25—H and N11—H (with a difference of only 1.5 kcal/mol between the two). The energy for N29—H is higher (10 kcal/mol), and it is much higher for N14—H (~30 kcal/mol). It seems that for the investigated structures, the calculated energies are much better suited for predicting protonation sequences than the ESP potentials.

### 3.3. ADMET Analysis

**Figure 23:**
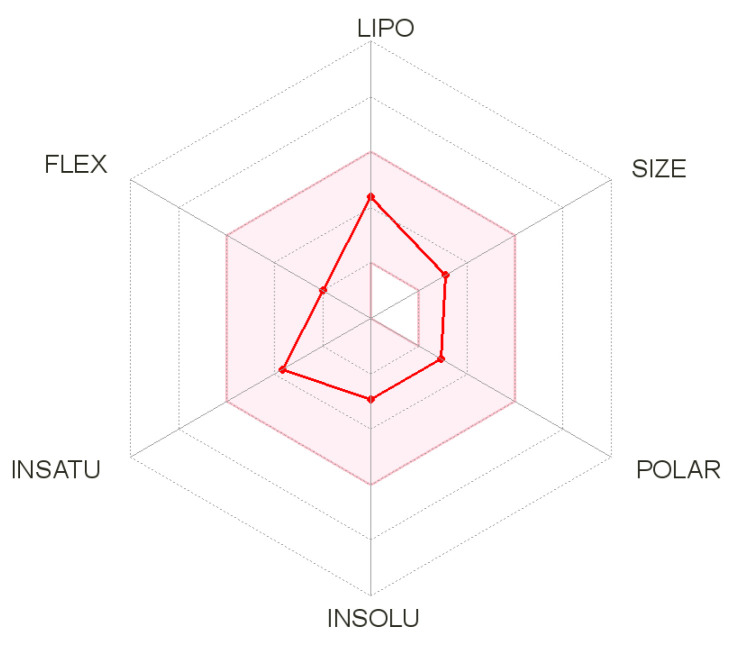
Bioavailability radars for compound 1. Pink zone—lipophilicity (LIPO) values are within the range −0.7 < XlogP3 < +5.0; molecular weight (SIZE) values are 150 g/mol < MW < 500 g/mol; polarity (POLAR) values are 20 Å^2^ < TPSA < 130 Å^2^; insolubility (INSOLU) values are 0 < logS < 6; insaturation (INSATU) values are 0.25 < Fraction Csp3 < 1; flexibility (FLEX) values are 0 < Num. rotatable bonds < 9.

**Figure 24:**
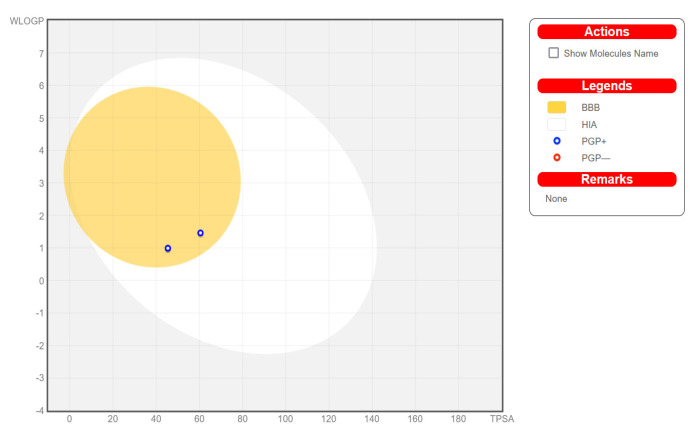
Boiled-egg diagram for all compounds.

The bioavailability radars for all the studied compounds are very similar (Figure 23 for compound **1**). The pink-colored zone on the bioavailability radar (SwissADME) presented the optimal range for each property, indicating the drug-likeness of a molecule. All the compounds meet the rules of Lipinski [32], Ghose [33], Egan [34], Veber [35], and Muegge [36]. All the compounds were found to be highly absorbed in the gastrointestinal tract, making them effective drugs (Figure 24). An important element is that, having high activity towards the H3 receptor, it crosses the blood–brain barrier. All of the studied compounds are not inhibitors of the CYP3A4 isoenzyme, which is largely responsible for the metabolism and elimination of most clinically used drugs [37]. Negative logKp values indicate that the compounds are not available through the skin.

Servis ProTox II classified the compounds **1**, **3,** and **5** into toxicity class 4 (harmful if swallowed), with a predicted LD50 of 1000 mg/kg. Compounds containing a sulfur atom (**2** and **4**) are in toxicity class 3 (toxic if swallowed), with a predicted LD50 of 300 mg/kg.

## 4. Conclusions

Seven new crystal structures (histamine H3 antagonists) were determined, including two polymorphs and one hydrate of the same compound. Interestingly, polymorphs **6** and **7** were both obtained from the same batch of crystallization. The two main factors differentiating the conformation of the studied molecules are as follows: (i) chain conformation, defined by the torsion angle N11–C3, and (ii) conformation at N14, defined by the angle between the N14–C21 bond and the C13–N14–C15 plane. The variability in the latter parameter is probably due to the intermolecular interactions occurring in the crystal structures, which is confirmed by QM calculations. The ADME analysis confirmed that the tested compounds are good drug candidates. For thiazole derivatives (compounds **2** and **4**), which show higher activity (as non-imidazole antagonists of histamine H3) than their oxazole analogues, the relative position of the aromatic bicyclic system and the piperazine ring is slightly different to that of oxazoles, which may affect their biological activity.

## Figures and Tables

**Table 1:** Crystal data, data collection and refinement details.

Compound	1	2	3	4	5	6	7
Chemical formula	C_13_H_20_N_4_O^2+^·2(Br^−^)·H_2_O	2(C_13_H_19_N_4_S^+^)·C_3_H_7_NO·2(Br^−^)	C_13_H_19.50_N_4_O_1.5_ + 1.5(Br^1.5-^)·0.25(C_3_H_7_NO)·H_2_O	C_13_H_19_N_4_S^+^·Br^−^	C_13_H_20_N_4_O^2+^·2(Br^−^)·H_2_O	C_13_H_19_N_4_O^+^·Br^−^	C_13_H_19_N_4_O^+^·Br^−^
*M*_r_	426.15	759.66	404.22	343.28	426.15	327.22	327.22
Space group	*P*-1	*I*2/*a*	*C*2/*c*	*Pbcn*	*Pbca*	*P*-1	*P*2_1_
Temperature (K)	100	100	100	100	102	100	100
*a*, *b*, *c* (Å)	5.9496 (1), 12.4570 (2), 12.8656 (1)	22.2087 (3), 7.5519 (1), 19.9225 (2)	51.1351 (4), 9.36026 (8), 7.19352 (8)	19.2189 (2), 20.6172 (2), 7.4439 (1)	7.4967 (1), 12.2531 (2), 36.9527 (6)	7.2634 (3), 11.1261 (5), 18.5460 (7)	8.10852 (3), 7.06025 (3), 12.41650 (7)
α, β, γ (°)	112.445 (2), 95.687 (2), 103.040 (2)	90, 92.368 (1), 90	90, 93.8822 (9), 90	90, 90, 90	90, 90, 90	80.561 (3), 80.848 (3), 76.840 (3)	90, 92.2991 (4), 90
*V* (Å^3^)	840.20 (3)	3338.51 (7)	3435.19 (6)	2949.57 (6)	3394.40 (9)	1428.04 (10)	710.25 (1)
*Z*	2	4	8	8	8	4	2
µ (mm^−1^)	6.21	4.56	4.74	5.06	6.15	3.92	3.94
No. of measured, independent and observed [*I* > 2σ(*I*)] reflections	87,115, 3443, 3276	20,179, 3404, 3251	4702, 4702, 4462	31,570, 3078, 2815	6678, 6678, 6025	39365, 6692, 6224	69,597, 2988, 2969
*R*[*F*^2^ > 2σ(*F*^2^)], *wR*(*F*^2^), *S*	0.039, 0.115, 1.13	0.025, 0.064, 1.07	0.046, 0.146, 1.05	0.020, 0.050, 1.04	0.071, 0.216, 1.08	0.080, 0.217, 1.10	0.033, 0.083, 1.05
No. of reflections	3443	3404	4702	3078	6678	6692	2988
No. of parameters	203	219	179	176	210	348	177
No. of restraints	0	0	0	0	3	0	1
Δ_max_, Δ_min_ (e Å^−3^)	1.43, −1.12	0.38, −0.43	1.07, −0.85	0.31, −0.41	1.87, −1.38	2.13, −2.22	2.24, −0.38
Absolute structure parameter	–	–	–	–	–	–	0.051 (10)

**Table 2:** Strong hydrogen-bond geometry (Å, º) for **1.** Symmetry codes: (i) *x*−1, *y*, *z*.

*D*—H···*A*	*D*—H	H···*A*	*D*···*A*	*D*—H···*A*
O1—H1*D*···Br2	0.83 (5)	2.47 (5)	3.263 (3)	161 (5)
O1—H1*E*···Br2^i^	0.74 (6)	2.85 (5)	3.436 (3)	138 (6)
N11—H11···Br1	0.90 (4)	2.27 (4)	3.169 (3)	174 (4)
N26—H26···O1	0.83 (5)	1.84 (5)	2.666 (4)	176 (6)

**Table 3:** Strong hydrogen-bond geometry (Å, º) for **2**.

*D*—H···*A*	*D*—H	H···*A*	*D*···*A*	*D*—H···*A*
N11—H11···Br1	0.88 (2)	2.31 (2)	3.1844 (15)	172 (2)

**Table 4:** Strong hydrogen-bond geometry (Å, º) for **3.** Symmetry codes: (i) −*x*+1, −*y*+1, −*z*+2.

*D*—H···*A*	*D*—H	H···*A*	*D*···*A*	*D*—H···*A*
N11—H11···Br1	0.98 (4)	2.28 (4)	3.227 (2)	163 (3)
N25—H25···N25^i^	0.88	1.81	2.684 (4)	176

**Table 5:** Strong hydrogen-bond geometry (Å, º) for **4**.

*D*—H···*A*	*D*—H	H···*A*	*D*···*A*	*D*—H···*A*
N11—H11···Br1	0.90 (2)	2.29 (2)	3.1903 (12)	174.0 (14)

**Table 6:** Strong hydrogen-bond geometry (Å, º) for **5**.

*D*—H···*A*	*D*—H	H···*A*	*D*···*A*	*D*—H···*A*
O1—H1*D*···Br2^i^	0.85 (8)	2.44 (8)	3.285 (6)	169 (8)
O1—H1*E*···Br1^ii^	0.86 (9)	2.53 (10)	3.342 (6)	159 (9)
N11—H11···Br1	0.85 (8)	2.34 (8)	3.184 (6)	172 (7)
N27—H27···Br2	0.83 (11)	2.41 (10)	3.232 (6)	169 (9)

Symmetry codes: (i) *x*−1/2, −*y*+1/2, −*z*+1.

**Table 7:** Strong hydrogen-bond geometry (Å, º) for **6**.

*D*—H···*A*	*D*—H	H···*A*	*D*···*A*	*D*—H···*A*
N11*A*—H11*A*···Br1*A*	1.00	2.20	3.189 (7)	169
N11*B*—H11*B*···Br1*B*	1.00	2.20	3.194 (7)	172

**Table 8:** Strong hydrogen-bond geometry (Å, º) for **7**.

*D*—H···*A*	*D*—H	H···*A*	*D*···*A*	*D*—H···*A*
N11—H11···Br1	1.06 (5)	2.15 (5)	3.180 (3)	163 (4)

**Table 9:** Energy of the optimized geometry of molecule 3 in the following different mono-protonated states: at N25, N29 and N11 nitrogen atoms, and CHELPG ESP fitted atomic charges for the optimized molecule 3 in unprotonated state.

Atoms	Energy (kcal/mol)	ESP Charges
N29	10.1	−0.69
N25	0	−0.61
N14	29.0	−0.19
N11	1.5	−0.53

## Data Availability

CCDC 2103464, 2103684, 2103707, 2104488, 2104517, 2105163, 2106627 contain the supplementary crystallographic data for this paper. The data are provided free of charge by The Cambridge Crystallographic Data Centre via www.ccdc.cam.ac.uk/structures.

## Supplementary Materials

The following are available online at https://www.mdpi.com/article/10.3390/ma14227094/s1, Cif files containing all crystallographic data.
