# Supplementary material for: Structures 4-n-propyl Piperazines as Non-Imidazole Histamine H3 Antagonists
Source: Materials (Basel). 2021 Nov 22;14(22):7094. doi: 10.3390/ma14227094 (PMC8621284; doi:10.3390/ma14227094)
Supplement: Supplementary file 1 [file materials-14-07094-s001.zip › 2103707_7_new_acc_depo-data_7_new_file002.html]

checkCIF/PLATON report


```
No syntax errors found.                               CIF dictionary  
Please wait while processing ....                     Interpreting this report
```

**Datablock: 7\_new**


---

|  |  |  |
| --- | --- | --- |
| Bond precision: | C-C = 0.0050 A | Wavelength=1.54184 |

|  |  |  |  |
| --- | --- | --- | --- |
| Cell: | a=8.10852(3) | b=7.06025(3) | c=12.41650(7) |
|  | alpha=90 | beta=92.2991(4) | gamma=90 |
| Temperature: | 100 K |  |  |

|  |  |  |
| --- | --- | --- |
|  | Calculated | Reported |
| Volume | 710.250(6) | 710.250(6) |
| Space group | P 21 | P 21 |
| Hall group | P 2yb | P 2yb |
| Moiety formula | C13 H19 N4 O, Br | C13 H19 N4 O 1+, Br 1- |
| Sum formula | C13 H19 Br N4 O | C13 H19 Br N4 O |
| Mr | 327.22 | 327.22 |
| Dx,g cm-3 | 1.530 | 1.530 |
| Z | 2 | 2 |
| Mu (mm-1) | 3.936 | 3.936 |
| F000 | 336.0 | 336.0 |
| F000' | 335.40 |  |
| h,k,lmax | 10,8,15 | 10,8,15 |
| Nref | 3058[ 1658] | 2988 |
| Tmin,Tmax | 0.765,0.899 | 0.216,1.000 |
| Tmin' | 0.039 |  |

|  |  |
| --- | --- |
| Correction method= # Reported T Limits: Tmin=0.216 Tmax=1.000 AbsCorr = GAUSSIAN |  |

|  |  |
| --- | --- |
| Data completeness= 1.80/0.98 | Theta(max)= 78.780 |

|  |  |
| --- | --- |
| R(reflections)= 0.0333( 2969) | wR2(reflections)= 0.0830( 2988) |
| |  |  | | --- | --- | | S = 1.054 | Npar= 177 | |

---

```
The following ALERTS were generated. Each ALERT has the format
       test-name_ALERT_alert-type_alert-level.
Click on the hyperlinks for more details of the test.


---

Alert level B
PLAT094_ALERT_2_B Ratio of Maximum / Minimum Residual Density ....       5.83 Report


---

Alert level C
PLAT353_ALERT_3_C Long   N-H (N0.87,N1.01A)  N11      - H11      .       1.06 Ang.  
PLAT971_ALERT_2_C Check Calcd Resid. Dens.  0.80A   From Br1             2.30 eA-3  
PLAT971_ALERT_2_C Check Calcd Resid. Dens.  0.97A   From Br1             1.74 eA-3  
PLAT987_ALERT_1_C The Flack x is >> 0 -  Do a BASF/TWIN Refinement     Please Check 


---

Alert level G
PLAT013_ALERT_1_G N.O.K.   _shelx_hkl_checksum Found in CIF ......     Please Check 
PLAT033_ALERT_4_G Flack x Value Deviates > 3.0 * sigma from Zero .      0.051 Note  
PLAT063_ALERT_4_G Crystal Size Possibly too Large for Beam Size ..       0.81 mm    
PLAT142_ALERT_4_G s.u. on b - Axis Small or Missing ..............    0.00003 Ang.  
PLAT143_ALERT_4_G s.u. on c - Axis Small or Missing ..............    0.00007 Ang.  
PLAT145_ALERT_4_G s.u. on beta     Small or Missing ..............     0.0004 Degree
PLAT398_ALERT_2_G Deviating  C-O-C    Angle From 120 for O22            102.9 Degree
PLAT912_ALERT_4_G Missing # of FCF Reflections Above STh/L=  0.600         13 Note  
PLAT978_ALERT_2_G Number C-C Bonds with Positive Residual Density.          0 Info  


---

   0 ALERT level A = Most likely a serious problem - resolve or explain
   1 ALERT level B = A potentially serious problem, consider carefully
   4 ALERT level C = Check. Ensure it is not caused by an omission or oversight
   9 ALERT level G = General information/check it is not something unexpected

   2 ALERT type 1 CIF construction/syntax error, inconsistent or missing data
   5 ALERT type 2 Indicator that the structure model may be wrong or deficient
   1 ALERT type 3 Indicator that the structure quality may be low
   6 ALERT type 4 Improvement, methodology, query or suggestion
   0 ALERT type 5 Informative message, check
```

---

---

It is advisable to attempt to resolve as many as possible of the alerts in all categories. Often the minor alerts point to easily fixed oversights, errors and omissions in your CIF or refinement strategy, so attention to these fine details can be worthwhile. In order to resolve some of the more serious problems it may be necessary to carry out additional measurements or structure refinements. However, the purpose of your study may justify the reported deviations and the more serious of these should normally be commented upon in the discussion or experimental section of a paper or in the "special\_details" fields of the CIF. checkCIF was carefully designed to identify outliers and unusual parameters, but every test has its limitations and alerts that are not important in a particular case may appear. Conversely, the absence of alerts does not guarantee there are no aspects of the results needing attention. It is up to the individual to critically assess their own results and, if necessary, seek expert advice. **Publication of your CIF in IUCr journals** A basic structural check has been run on your CIF. These basic checks will be run on all CIFs submitted for publication in IUCr journals (*Acta Crystallographica*, *Journal of Applied Crystallography*, *Journal of Synchrotron Radiation*); however, if you intend to submit to *Acta Crystallographica Section C* or *E* or *IUCrData*, you should make sure that full publication checks are run on the final version of your CIF prior to submission. **Publication of your CIF in other journals** Please refer to the *Notes for Authors* of the relevant journal for any special instructions relating to CIF submission. |

---

**PLATON version of 13/07/2021; check.def file version of 13/07/2021**

|  |
| --- |
| **Datablock 7\_new** - ellipsoid plot |
|  |

---

 Download CIF editor (publCIF) from the IUCr   
 Download CIF editor (enCIFer) from the CCDC   
 Test a new CIF entry 
