# Supplementary material for: Structures 4-n-propyl Piperazines as Non-Imidazole Histamine H3 Antagonists
Source: Materials (Basel). 2021 Nov 22;14(22):7094. doi: 10.3390/ma14227094 (PMC8621284; doi:10.3390/ma14227094)
Supplement: Supplementary file 1 [file materials-14-07094-s001.zip › 2104488_2_new_acc_depo-data_2_new_file002.html]

checkCIF/PLATON report


```
No syntax errors found.                               CIF dictionary  
Please wait while processing ....                     Interpreting this report
```

**Datablock: 2\_new**


---

|  |  |  |
| --- | --- | --- |
| Bond precision: | C-C = 0.0030 A | Wavelength=1.54184 |

|  |  |  |  |
| --- | --- | --- | --- |
| Cell: | a=22.2087(3) | b=7.5519(1) | c=19.9225(2) |
|  | alpha=90 | beta=92.368(1) | gamma=90 |
| Temperature: | 100 K |  |  |

|  |  |  |
| --- | --- | --- |
|  | Calculated | Reported |
| Volume | 3338.51(7) | 3338.51(7) |
| Space group | I 2/a | I 2/a |
| Hall group | -I 2ya | -I 2ya |
| Moiety formula | 2(C13 H19 N4 S), C3 H7 N O, 2(Br) | 2(C13 H19 N4 S 1+), C3 H7 N O, 2(Br 1-) |
| Sum formula | C29 H45 Br2 N9 O S2 | C29 H45 Br2 N9 O S2 |
| Mr | 759.66 | 759.66 |
| Dx,g cm-3 | 1.511 | 1.511 |
| Z | 4 | 4 |
| Mu (mm-1) | 4.557 | 4.557 |
| F000 | 1568.0 | 1568.0 |
| F000' | 1568.40 |  |
| h,k,lmax | 28,9,25 | 26,9,24 |
| Nref | 3619 | 3404 |
| Tmin,Tmax | 0.678,0.913 | 0.228,1.000 |
| Tmin' | 0.056 |  |

|  |  |
| --- | --- |
| Correction method= # Reported T Limits: Tmin=0.228 Tmax=1.000 AbsCorr = MULTI-SCAN |  |

|  |  |
| --- | --- |
| Data completeness= 0.941 | Theta(max)= 79.150 |

|  |  |
| --- | --- |
| R(reflections)= 0.0254( 3251) | wR2(reflections)= 0.0642( 3404) |
| |  |  | | --- | --- | | S = 1.072 | Npar= 219 | |

---

```
The following ALERTS were generated. Each ALERT has the format
       test-name_ALERT_alert-type_alert-level.
Click on the hyperlinks for more details of the test.


---

Alert level B
PLAT934_ALERT_3_B Number of (Iobs-Icalc)/Sigma(W) > 10 Outliers ..          2 Check 


---

Alert level C
PLAT911_ALERT_3_C Missing FCF Refl Between Thmin & STh/L=    0.600          2 Report


---

Alert level G
PLAT013_ALERT_1_G N.O.K.   _shelx_hkl_checksum Found in CIF ......     Please Check 
PLAT063_ALERT_4_G Crystal Size Possibly too Large for Beam Size ..       0.62 mm    
PLAT083_ALERT_2_G SHELXL Second Parameter in WGHT  Unusually Large       9.47 Why ? 
PLAT128_ALERT_4_G Alternate Setting for Input Space Group  I2/a          I2/c Note  
PLAT300_ALERT_4_G Atom Site Occupancy of O31        Constrained at        0.5 Check

And 10 other PLAT300 Alerts

PLAT300_ALERT_4_G Atom Site Occupancy of C31        Constrained at        0.5 Check 
PLAT300_ALERT_4_G Atom Site Occupancy of C32        Constrained at        0.5 Check 
PLAT300_ALERT_4_G Atom Site Occupancy of C33        Constrained at        0.5 Check 
PLAT300_ALERT_4_G Atom Site Occupancy of H31        Constrained at        0.5 Check 
PLAT300_ALERT_4_G Atom Site Occupancy of H32A       Constrained at        0.5 Check 
PLAT300_ALERT_4_G Atom Site Occupancy of H32B       Constrained at        0.5 Check 
PLAT300_ALERT_4_G Atom Site Occupancy of H32C       Constrained at        0.5 Check 
PLAT300_ALERT_4_G Atom Site Occupancy of H33A       Constrained at        0.5 Check 
PLAT300_ALERT_4_G Atom Site Occupancy of H33B       Constrained at        0.5 Check 
PLAT300_ALERT_4_G Atom Site Occupancy of H33C       Constrained at        0.5 Check

PLAT302_ALERT_4_G Anion/Solvent/Minor-Residue Disorder (Resd  2  )        80% Note  
PLAT789_ALERT_4_G Atoms with Negative _atom_site_disorder_group  #         11 Check 
PLAT912_ALERT_4_G Missing # of FCF Reflections Above STh/L=  0.600        190 Note  
PLAT950_ALERT_5_G Calculated (ThMax) and CIF-Reported Hmax Differ           2 Units 
PLAT956_ALERT_1_G Calculated (ThMax) and Actual (FCF) Hmax Differ           2 Units 
PLAT978_ALERT_2_G Number C-C Bonds with Positive Residual Density.          4 Info  


---

   0 ALERT level A = Most likely a serious problem - resolve or explain
   1 ALERT level B = A potentially serious problem, consider carefully
   1 ALERT level C = Check. Ensure it is not caused by an omission or oversight
  21 ALERT level G = General information/check it is not something unexpected

   2 ALERT type 1 CIF construction/syntax error, inconsistent or missing data
   2 ALERT type 2 Indicator that the structure model may be wrong or deficient
   2 ALERT type 3 Indicator that the structure quality may be low
  16 ALERT type 4 Improvement, methodology, query or suggestion
   1 ALERT type 5 Informative message, check
```

---

---

It is advisable to attempt to resolve as many as possible of the alerts in all categories. Often the minor alerts point to easily fixed oversights, errors and omissions in your CIF or refinement strategy, so attention to these fine details can be worthwhile. In order to resolve some of the more serious problems it may be necessary to carry out additional measurements or structure refinements. However, the purpose of your study may justify the reported deviations and the more serious of these should normally be commented upon in the discussion or experimental section of a paper or in the "special\_details" fields of the CIF. checkCIF was carefully designed to identify outliers and unusual parameters, but every test has its limitations and alerts that are not important in a particular case may appear. Conversely, the absence of alerts does not guarantee there are no aspects of the results needing attention. It is up to the individual to critically assess their own results and, if necessary, seek expert advice. **Publication of your CIF in IUCr journals** A basic structural check has been run on your CIF. These basic checks will be run on all CIFs submitted for publication in IUCr journals (*Acta Crystallographica*, *Journal of Applied Crystallography*, *Journal of Synchrotron Radiation*); however, if you intend to submit to *Acta Crystallographica Section C* or *E* or *IUCrData*, you should make sure that full publication checks are run on the final version of your CIF prior to submission. **Publication of your CIF in other journals** Please refer to the *Notes for Authors* of the relevant journal for any special instructions relating to CIF submission. |

---

**PLATON version of 13/07/2021; check.def file version of 13/07/2021**

|  |
| --- |
| **Datablock 2\_new** - ellipsoid plot |
|  |

---

 Download CIF editor (publCIF) from the IUCr   
 Download CIF editor (enCIFer) from the CCDC   
 Test a new CIF entry 
