# Supplementary material for: Structures 4-n-propyl Piperazines as Non-Imidazole Histamine H3 Antagonists
Source: Materials (Basel). 2021 Nov 22;14(22):7094. doi: 10.3390/ma14227094 (PMC8621284; doi:10.3390/ma14227094)
Supplement: Supplementary file 1 [file materials-14-07094-s001.zip › 2106627_3_new_acc_depo-data_3_new_file002.html]

checkCIF/PLATON report


```
No syntax errors found.                               CIF dictionary  
Please wait while processing ....                     Interpreting this report
```

**Datablock: 3\_new**


---

|  |  |  |
| --- | --- | --- |
| Bond precision: | C-C = 0.0043 A | Wavelength=1.54184 |

|  |  |  |  |
| --- | --- | --- | --- |
| Cell: | a=51.1351(4) | b=9.36026(8) | c=7.19352(8) |
|  | alpha=90 | beta=93.8822(9) | gamma=90 |
| Temperature: | 100 K |  |  |

|  |  |  |
| --- | --- | --- |
|  | Calculated | Reported |
| Volume | 3435.19(5) | 3435.19(6) |
| Space group | C 2/c | C 2/c |
| Hall group | -C 2yc | -C 2yc |
| Moiety formula | 2(C13 H19.50 N4 O), 2(Br) [+ solvent] | C13 H19.50 N4 O 1.5+, 1.5(Br 1.5-), 0.25(C3 H7 N O), H2 O |
| Sum formula | C26 H39 Br2 N8 O2 [+ solvent] | C13.75 H23.50 Br1.50 N4.25 O2.25 |
| Mr | 655.45 | 404.22 |
| Dx,g cm-3 | 1.267 | 1.563 |
| Z | 4 | 8 |
| Mu (mm-1) | 3.255 | 4.744 |
| F000 | 1348.0 | 1348.0 |
| F000' | 1345.59 |  |
| h,k,lmax | 64,11,9 | 64,11,9 |
| Nref | 3697 | 4702 |
| Tmin,Tmax | 0.705,0.752 | 0.451,0.847 |
| Tmin' | 0.098 |  |

|  |  |
| --- | --- |
| Correction method= # Reported T Limits: Tmin=0.451 Tmax=0.847 AbsCorr = ANALYTICAL |  |

|  |  |
| --- | --- |
| Data completeness= 1.272 | Theta(max)= 78.820 |

|  |  |
| --- | --- |
| R(reflections)= 0.0464( 4462) | wR2(reflections)= 0.1462( 4702) |
| |  |  | | --- | --- | | S = 1.052 | Npar= 179 | |

---

```
The following ALERTS were generated. Each ALERT has the format
       test-name_ALERT_alert-type_alert-level.
Click on the hyperlinks for more details of the test.


---

Alert level C
PLAT911_ALERT_3_C Missing FCF Refl Between Thmin & STh/L=    0.600          4 Report
PLAT918_ALERT_3_C Reflection(s) with I(obs) much Smaller I(calc) .          1 Check 


---

Alert level G
FORMU01_ALERT_1_G  There is a discrepancy between the atom counts in the
            _chemical_formula_sum and _chemical_formula_moiety. This is
            usually due to the moiety formula being in the wrong format.
            Atom count from _chemical_formula_sum:   C13.75 H23.5 Br1.5 N4.25 O2.2
            Atom count from _chemical_formula_moiety:C13.75 H23.25 Br1.5 N4.25 O2.
FORMU01_ALERT_2_G  There is a discrepancy between the atom counts in the
            _chemical_formula_sum and the formula from the _atom_site* data.
            Atom count from _chemical_formula_sum:C13.75 H23.5 Br1.5 N4.25 O2.25
            Atom count from the _atom_site data:  C13 H19.5 Br1 N4 O1
CELLZ01_ALERT_1_G Difference between formula and atom_site contents detected.
CELLZ01_ALERT_1_G ALERT: Large difference may be due to a
            symmetry error - see SYMMG tests
           From the CIF: _cell_formula_units_Z    8
           From the CIF: _chemical_formula_sum  C13.75 H23.50 Br1.50 N4.25 O2.25
           TEST: Compare cell contents of formula and atom_site data

           atom    Z*formula  cif sites diff
           C        110.00    104.00    6.00
           H        188.00    156.00   32.00
           Br        12.00      8.00    4.00
           N         34.00     32.00    2.00
           O         18.00      8.00   10.00
PLAT007_ALERT_5_G Number of Unrefined Donor-H Atoms ..............          1 Report
PLAT041_ALERT_1_G Calc. and Reported SumFormula    Strings  Differ     Please Check 
PLAT042_ALERT_1_G Calc. and Reported Moiety Formula Strings Differ     Please Check 
PLAT045_ALERT_1_G Calculated and Reported Z Differ by a Factor ...       0.50 Check 
PLAT051_ALERT_1_G Mu(calc) and Mu(CIF) Ratio Differs from 1.0 by .      31.38 %     
PLAT072_ALERT_2_G SHELXL First  Parameter in WGHT  Unusually Large       0.10 Report
PLAT083_ALERT_2_G SHELXL Second Parameter in WGHT  Unusually Large       6.10 Why ? 
PLAT142_ALERT_4_G s.u. on b - Axis Small or Missing ..............    0.00008 Ang.  
PLAT300_ALERT_4_G Atom Site Occupancy of H25        Constrained at        0.5 Check 
PLAT304_ALERT_4_G Non-Integer Number of Atoms in ..... (Resd  1  )      37.50 Check 
PLAT398_ALERT_2_G Deviating  C-O-C    Angle From 120 for O22            103.1 Degree
PLAT605_ALERT_4_G Largest Solvent Accessible VOID in the Structure        321 A**3  
PLAT869_ALERT_4_G ALERTS Related to the Use of SQUEEZE Suppressed           ! Info  
PLAT870_ALERT_4_G ALERTS Related to Twinning Effects Suppressed ..          ! Info  
PLAT883_ALERT_1_G No Info/Value for _atom_sites_solution_primary .     Please Do !  
PLAT910_ALERT_3_G Missing # of FCF Reflection(s) Below Theta(Min).          1 Note  
PLAT912_ALERT_4_G Missing # of FCF Reflections Above STh/L=  0.600         73 Note  
PLAT941_ALERT_3_G Average HKL Measurement Multiplicity ...........        1.3 Low   


---

   0 ALERT level A = Most likely a serious problem - resolve or explain
   0 ALERT level B = A potentially serious problem, consider carefully
   2 ALERT level C = Check. Ensure it is not caused by an omission or oversight
  22 ALERT level G = General information/check it is not something unexpected

   8 ALERT type 1 CIF construction/syntax error, inconsistent or missing data
   4 ALERT type 2 Indicator that the structure model may be wrong or deficient
   4 ALERT type 3 Indicator that the structure quality may be low
   7 ALERT type 4 Improvement, methodology, query or suggestion
   1 ALERT type 5 Informative message, check
```

---

---

It is advisable to attempt to resolve as many as possible of the alerts in all categories. Often the minor alerts point to easily fixed oversights, errors and omissions in your CIF or refinement strategy, so attention to these fine details can be worthwhile. In order to resolve some of the more serious problems it may be necessary to carry out additional measurements or structure refinements. However, the purpose of your study may justify the reported deviations and the more serious of these should normally be commented upon in the discussion or experimental section of a paper or in the "special\_details" fields of the CIF. checkCIF was carefully designed to identify outliers and unusual parameters, but every test has its limitations and alerts that are not important in a particular case may appear. Conversely, the absence of alerts does not guarantee there are no aspects of the results needing attention. It is up to the individual to critically assess their own results and, if necessary, seek expert advice. **Publication of your CIF in IUCr journals** A basic structural check has been run on your CIF. These basic checks will be run on all CIFs submitted for publication in IUCr journals (*Acta Crystallographica*, *Journal of Applied Crystallography*, *Journal of Synchrotron Radiation*); however, if you intend to submit to *Acta Crystallographica Section C* or *E* or *IUCrData*, you should make sure that full publication checks are run on the final version of your CIF prior to submission. **Publication of your CIF in other journals** Please refer to the *Notes for Authors* of the relevant journal for any special instructions relating to CIF submission. |

---

**PLATON version of 13/07/2021; check.def file version of 13/07/2021**

|  |
| --- |
| **Datablock 3\_new** - ellipsoid plot |
|  |

---

 Download CIF editor (publCIF) from the IUCr   
 Download CIF editor (enCIFer) from the CCDC   
 Test a new CIF entry 
