# Supplementary material for: Structures 4-n-propyl Piperazines as Non-Imidazole Histamine H3 Antagonists
Source: Materials (Basel). 2021 Nov 22;14(22):7094. doi: 10.3390/ma14227094 (PMC8621284; doi:10.3390/ma14227094)
Supplement: Supplementary file 1 [file materials-14-07094-s001.zip › 2103684_6_new_acc_depo-data_6_new_file002.html]

checkCIF/PLATON report


```
No syntax errors found.                               CIF dictionary  
Please wait while processing ....                     Interpreting this report
```

**Datablock: 6\_new**


---

|  |  |  |
| --- | --- | --- |
| Bond precision: | C-C = 0.0116 A | Wavelength=1.54184 |

|  |  |  |  |
| --- | --- | --- | --- |
| Cell: | a=7.2634(3) | b=11.1261(5) | c=18.5460(7) |
|  | alpha=80.561(3) | beta=80.848(3) | gamma=76.840(3) |
| Temperature: | 100 K |  |  |

|  |  |  |
| --- | --- | --- |
|  | Calculated | Reported |
| Volume | 1428.04(10) | 1428.04(10) |
| Space group | P -1 | P -1 |
| Hall group | -P 1 | -P 1 |
| Moiety formula | C13 H19 N4 O, Br | C13 H19 N4 O 1+, Br 1- |
| Sum formula | C13 H19 Br N4 O | C13 H19 Br1 N4 O1 |
| Mr | 327.22 | 327.22 |
| Dx,g cm-3 | 1.522 | 1.522 |
| Z | 4 | 4 |
| Mu (mm-1) | 3.915 | 3.915 |
| F000 | 672.0 | 672.0 |
| F000' | 670.80 |  |
| h,k,lmax | 9,14,23 | 9,13,23 |
| Nref | 6153 | 6692 |
| Tmin,Tmax | 0.541,0.806 | 0.191,0.856 |
| Tmin' | 0.008 |  |

|  |  |
| --- | --- |
| Correction method= # Reported T Limits: Tmin=0.191 Tmax=0.856 AbsCorr = ANALYTICAL |  |

|  |  |
| --- | --- |
| Data completeness= 1.088 | Theta(max)= 78.570 |

|  |  |
| --- | --- |
| R(reflections)= 0.0801( 6224) | wR2(reflections)= 0.2170( 6692) |
| |  |  | | --- | --- | | S = 1.098 | Npar= 348 | |

---

```
The following ALERTS were generated. Each ALERT has the format
       test-name_ALERT_alert-type_alert-level.
Click on the hyperlinks for more details of the test.


---

Alert level B
CRYSS02_ALERT_3_B  The value of _exptl_crystal_size_max is > 1.0
   Maximum crystal size given =   1.222


---

Alert level C
PLAT341_ALERT_3_C Low Bond Precision on  C-C Bonds ...............    0.01162 Ang.  
PLAT906_ALERT_3_C Large K Value in the Analysis of Variance ......      2.971 Check 
PLAT911_ALERT_3_C Missing FCF Refl Between Thmin & STh/L=    0.600          3 Report
PLAT918_ALERT_3_C Reflection(s) with I(obs) much Smaller I(calc) .          4 Check 
PLAT939_ALERT_3_C Large Value of Not (SHELXL) Weight Optimized S .      12.68 Check 


---

Alert level G
PLAT007_ALERT_5_G Number of Unrefined Donor-H Atoms ..............          2 Report
PLAT063_ALERT_4_G Crystal Size Possibly too Large for Beam Size ..       1.22 mm    
PLAT083_ALERT_2_G SHELXL Second Parameter in WGHT  Unusually Large      13.96 Why ? 
PLAT093_ALERT_1_G No s.u.'s on H-positions, Refinement Reported as      mixed Check 
PLAT154_ALERT_1_G The s.u.'s on the Cell Angles are Equal ..(Note)      0.003 Degree
PLAT398_ALERT_2_G Deviating  C-O-C    Angle From 120 for O22A           102.9 Degree
PLAT398_ALERT_2_G Deviating  C-O-C    Angle From 120 for O22B           102.8 Degree
PLAT720_ALERT_4_G Number of Unusual/Non-Standard Labels ..........         14 Note  
PLAT790_ALERT_4_G Centre of Gravity not Within Unit Cell: Resd.  #          4 Note  
              Br                                                                
PLAT870_ALERT_4_G ALERTS Related to Twinning Effects Suppressed ..          ! Info  
PLAT910_ALERT_3_G Missing # of FCF Reflection(s) Below Theta(Min).          1 Note  
PLAT912_ALERT_4_G Missing # of FCF Reflections Above STh/L=  0.600        278 Note  
PLAT941_ALERT_3_G Average HKL Measurement Multiplicity ...........        1.1 Low   


---

   0 ALERT level A = Most likely a serious problem - resolve or explain
   1 ALERT level B = A potentially serious problem, consider carefully
   5 ALERT level C = Check. Ensure it is not caused by an omission or oversight
  13 ALERT level G = General information/check it is not something unexpected

   2 ALERT type 1 CIF construction/syntax error, inconsistent or missing data
   3 ALERT type 2 Indicator that the structure model may be wrong or deficient
   8 ALERT type 3 Indicator that the structure quality may be low
   5 ALERT type 4 Improvement, methodology, query or suggestion
   1 ALERT type 5 Informative message, check
```

---

---

It is advisable to attempt to resolve as many as possible of the alerts in all categories. Often the minor alerts point to easily fixed oversights, errors and omissions in your CIF or refinement strategy, so attention to these fine details can be worthwhile. In order to resolve some of the more serious problems it may be necessary to carry out additional measurements or structure refinements. However, the purpose of your study may justify the reported deviations and the more serious of these should normally be commented upon in the discussion or experimental section of a paper or in the "special\_details" fields of the CIF. checkCIF was carefully designed to identify outliers and unusual parameters, but every test has its limitations and alerts that are not important in a particular case may appear. Conversely, the absence of alerts does not guarantee there are no aspects of the results needing attention. It is up to the individual to critically assess their own results and, if necessary, seek expert advice. **Publication of your CIF in IUCr journals** A basic structural check has been run on your CIF. These basic checks will be run on all CIFs submitted for publication in IUCr journals (*Acta Crystallographica*, *Journal of Applied Crystallography*, *Journal of Synchrotron Radiation*); however, if you intend to submit to *Acta Crystallographica Section C* or *E* or *IUCrData*, you should make sure that full publication checks are run on the final version of your CIF prior to submission. **Publication of your CIF in other journals** Please refer to the *Notes for Authors* of the relevant journal for any special instructions relating to CIF submission. |

---

**PLATON version of 13/07/2021; check.def file version of 13/07/2021**

|  |
| --- |
| **Datablock 6\_new** - ellipsoid plot |
|  |

---

 Download CIF editor (publCIF) from the IUCr   
 Download CIF editor (enCIFer) from the CCDC   
 Test a new CIF entry 
